# Estimation of the crop evapotranspiration for Udham Singh Nagar district using modified Priestley-Taylor model and Landsat imagery

**DOI:** 10.1038/s41598-024-72299-x

**Published:** 2024-09-13

**Authors:** Anurag Satpathi, Abhishek Danodia, Salwan Ali Abed, Ajeet Singh Nain, Nadhir Al-Ansari, Rajeev Ranjan, Dinesh Kumar Vishwakarma, Amel Gacem, Lamjed Mansour, Krishna Kumar Yadav

**Affiliations:** 1 Department of Agrometeorology, College of Agriculture, Govind Ballabh Pant University of Agriculture and Technology, Pantnagar, Uttarakhand 263145 India; 2 Indian Institute of Remote Sensing, Indian Space Research Organisation, Bengaluru, India; 3 Department of Environment, College of Science, University of Al-Qadisiyah, Al-Qadisiyah, 58001 Iraq; 4 Department of Civil, Environmental, and Natural Resources Engineering, Lulea University of Technology, 97187 Lulea, Sweden; 5 Department of Irrigation and Drainage Engineering, Govind Ballabh Pant University of Agriculture and Technology, Pantnagar, Uttarakhand 263145 India; 6 Department of Physics, Faculty of Sciences, University of Skikda, 21000 Skikda, Algeria; 7 Department of Zoology, College of Science, King Saud University, 11451 Riyadh, Saudi Arabia; 8 Department of Environmental Science, Parul Institute of Applied Sciences, Parul University, Vadodara, Gujarat 391760 India; 9 Environmental and Atmospheric Sciences Research Group, Scientific Research Center, Al-Ayen University, Thi-Qar, Nasiriyah, 64001 Iraq

**Keywords:** Evapotranspiration, Modified Priestley-Taylor Landsat, Agrometeorology, Climate sciences, Environmental sciences, Hydrology

## Abstract

The main challenges for utilizing daily evapotranspiration (ET) estimation in the study area revolve around the need for accurate and reliable data inputs, as well as the interpretation of ET dynamics within the context of local agricultural practices and environmental conditions. Factors such as cloud cover, atmospheric aerosols, and variations in land cover pose challenges to the precise estimation of ET from remote sensing data. This research aimed to utilize Landsat 8 and 9 datasets from the 2022–23 period in the Udham Singh Nagar district to apply the modified Priestley-Taylor (MPT) model for estimating ET. An average ET was estimated 1.33, 1.57, 1.70, 2.99, and 3.20 mm day^−1^ with 0.29, 0.33, 0.41, 0.69, and 1.03 standard deviation for December, January, February, March, and April month, respectively. In the validation phase, a strong correlation was found between the evaporative fraction derived from MPT and that observed by lysimeter, with R^2^ = 0.71, mean biased error = 0.04 mm day^−1^, root mean squared error = 0.62 mm day^-1^ and agreement index of 0.914. These results collectively support the effectiveness of the MPT model in accurately estimating ET across Udham Singh Nagar district. In essence, this research not only confirms the MPT model’s capability in ET estimation but also offers detailed insights into the spatial and temporal fluctuations of energy fluxes and daily ET rates.

## Introduction

The amount of energy exchange taking place between the Earth’s surface and atmosphere can be determined through evapotranspiration (ET)^1,2^. The amount of ET depends on several factors such as land cover type, amount and duration of solar radiation, air temperature, speed of the wind above it etc^3,4^. About 90% of the total water used in agriculture is lost either by evaporation from soil or crop transpiration^2,5–7^. The actual crop evapotranspiration is an indicator of the water demand, crop stress, irrigation scheduling, drought and water budget study for crops and trees for healthy growth and development^7–10^. Hence, the accurate estimation of crop evapotranspiration is very important for both rainfed and irrigated agriculture for water management, drought analysis and crop management. The ET estimation can be done through lysimeter systems, eddy covariance towers, evaporation pans, Bowen ratio stations and scintillometer systems. All these methods mentioned here can measure ET in a relatively small area and difficult to extrapolate in both time and space. Apart from that these point measurements are only applicable to that area over which these are established or installed and there are many challenges in up scaling these values over a larger area. To overcome this problem several researchers developed empirical methods for estimating ET viz. Thornthwaite method^11^, Hargreaves method^12^, Penman equation^13^, Penman-Monteith^14^, Priestley Taylor (PT)^15^ and FAO 56 Penman–Monteith equation^16^ etc. The last method among all these methods is most widely used and adopted by the Food and Agriculture Organization (FAO)^4^. Again, all these methods depend on the local weather data and limited to that weather station area only. Hence these point-measured methodologies cannot be applied over extensive areas. Hence the need of remote sensing-based ET estimation for larger area comes into the picture. Among the several ET estimation methods, remote sensing can be regarded as the only technology that can accurately and economically provide ET amount at regional and global scale^17,18^.

Several researchers have developed many remote sensing-based ET models^19–21^ such as SEBAL: Surface energy balance algorithm for land^22,23^, S-SEBI: simplified surface energy balance index^24^, SEBS: Surface energy balance system^25^ and METRIC: Mapping evapotranspiration at high spatial resolution with Internalized calibration^17^. Among all the techniques developed through remote sensing for ET estimation, the energy balance method based on the modified Priestley-Taylor (MPT) approach has been widely used due to its simplicity and relatively low data demand. The MPT approach was first introduced by Priestley and Taylor in 1972^15^ and has since been modified to improve its accuracy and applicability in different regions and cropping systems^26,27^.

The triangle-based approach is firstly used by Jiang and Islam^28^ for satellite-based ET estimation. Since then, this method has been widely modified and adopted by scientists worldwide to estimate ET for different crops. Table 1 in this paper provides a detailed overview of relevant research literature, showcasing the various algorithms used for ET estimation across different scenarios and the corresponding outcomes. Notably, the table covers studies employing methods like the Ts-VI triangle method, Modified Priestley-Taylor model (MPT), Priestley-Taylor model (PT) and Penman–Monteith method (PM). It is essential to note that the models discussed in the table have been carefully calibrated and validated for specific regions and specific crop types.

**Table 1:** Different modified Priestley-Taylor based past studies and their results.

S. No	Author	Algorithm used	Target Problem	Results
1	Tang et al.^29^	Ts-VI triangle method	Quantify sensible heat flux	Helps to estimate regional surface ET accurately
2	Ding et al.^30^	Modified Priestley-Taylor	ET estimation over irrigated maize field	A good agreement was found between ET estimated by the model with observations
3	Qiu et al.^31^	Modified Priestley-Taylor	ET estimation in a rice–wheat rotation system	The model estimates ET for rice and winter wheat reasonably
4	Nikolaou et al.^32^	Modified Priestley-Taylor	Calibration of Priestley-Taylor (α) coefficient in Mediterranean greenhouse cucumbers	The proposed modified potential evapotranspiration model can be used as a practical method for irrigation scheduling
5	Ai and Yang^33^	Priestley-Taylor Model	Estimating Cotton Evapotranspiration under Plastic Mulch Condition	The estimated values agreed well with the measured values
6	Venturini et al.^34^	Modified Priestley-Taylor	Comparison among different modified Priestley and Taylor equations	Both atmospheric and surface variables should be jointly parameterized in order to obtain estimates with lower errors
7	Aschonitis et al.^35^	Priestley-Taylor method	To test the Priestley-Taylor method for the assessment of reference ET	The surface coverage of the Italian territory, with acceptable ± 10% difference
8	García et al.^36^	PT-JPL method	Improving regional estimates of actual evapotranspiration (λΕ) in water-limited regions	Both in-situ and satellite data produced satisfactory results for λΕ at the Sahelian savanna
9	Sumner and Jacobs^37^	Penman–Monteith and MPT	Estimating pasture evapotranspiration using different methods	The PM method was slightly less effective than the PT method
10	Yao et al.^38^	Modified Priestley-Taylor	MPT algorithm is used to estimate ET then validated	MPT algorithm is satisfactory in reproducing the inter-annual variability at flux tower sites

Building on these existing approaches, we propose a modified method for ET estimation based on the MPT framework. Our proposed method offers the advantage of accurately estimating ET not only for sugarcane crops but for all the crops over study area, without relying on any ground-based observed data, which could significantly benefit agricultural practices.

Remote sensing technology plays a crucial role in contemporary environmental studies by providing a comprehensive view of the Earth's surface and its dynamic processes^39,40^. Among the array of remote sensing platforms, the Landsat series stands out as an indispensable tool due to its long-term, consistent provision of high-quality data^41^. With the recent launch of Landsat 9, the capabilities of this iconic satellite constellation have been further augmented, promising enhanced insights into Earth’s land cover, land use, and environmental changes. Equipped with the Operational Land Imager (OLI) and Thermal Infrared Sensor (TIRS), Landsat 8 and 9 has facilitated numerous applications, including precision agriculture, forestry management, water management and urban planning^42,43^. Its multispectral capabilities, coupled with a revisit time of approximately 16 days, have enabled researchers to monitor changes in land cover with unprecedented detail and frequency. The availability of Landsat 8 and 9 data represents a transformative opportunity to deepen our understanding of evapotranspiration and water budgeting. Table 2 contains the characteristics of Landsat 8 and 9 datasets.

**Table 2:** Characteristics of Landsat 8–9 OLI/TIRS collection.

Pixel size	OLI multispectral bands (Band 1–7 and 9): 30 m
	OLI panchromatic band (Band 8): 15 m
	TIRS band (Band 10 and 11): 100 m (Resampled to 30 m)
Data characteristics	North up (MAP) orientation
	Universal transverse Mercator (UTM) map projection
	World Geodetic System (WGS) – 84 datum
	16-bit pixel values

In the realm of innovative methodologies, the surface temperature-vegetation index (Ts-VI) triangle method, a variant of the Modified Priestley-Taylor (MPT) approach, emerged through the pioneering work of Jiang and Islam in 1999 and 2001, later refined in 2003. The modified Priestley-Taylor model is simplified form of the Penman equation ^30,44^. The accuracy of the PT model depends mainly on the precise determination of the PT coefficient^15,45,46^. A distinguishing feature of the PT model lies in its exemption from the need for measuring sensible heat flux and computing the ratio of sensible heat flux to latent heat flux. Extensive investigations, led by scholars such as Khaldi et al.^47^ and Parlange and Katul^48^, advocate for the adept application of a PT coefficient value of 1.26, particularly conducive to vegetative expanses. The versatility of this model has been showcased in various studies, as evidenced by the works of Jiang and Islam^28^ and Yao et al.^38^. Leaf area index (LAI), vapor pressure deficit (VPD) and soil moisture content (θ) are the main factors, which can affect the PT coefficient^49,50^.

Therefore, the main objective of this research is to estimate crop evapotranspiration using the energy balance based modified Priestley-Taylor approach approaches for the Udham Singh Nagar district of Uttarakhand, India. To assess the accuracy of this approach a comparison is made between remotely sensed estimated ET values with measured ET data from lysimeter installed in the study region. The outcomes of this study will help to improve water management practices and increase crop yield in the region.

## Site description and data used

### Study area

**Fig.1:**
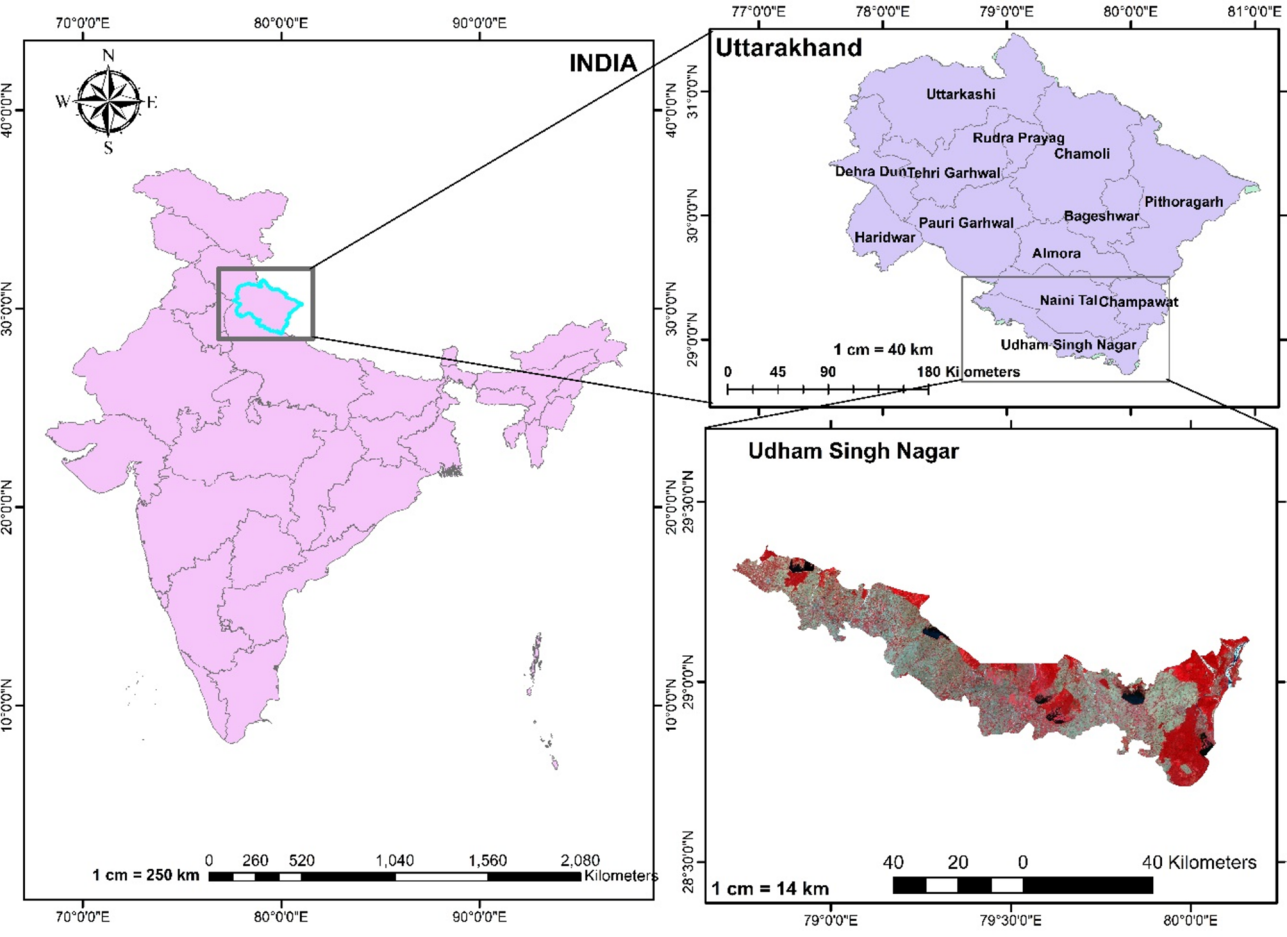
Location map of study area with standard false colour composite generated from Landsat 8 OLI data (23rd Nov 2022).

**Fig.2:**
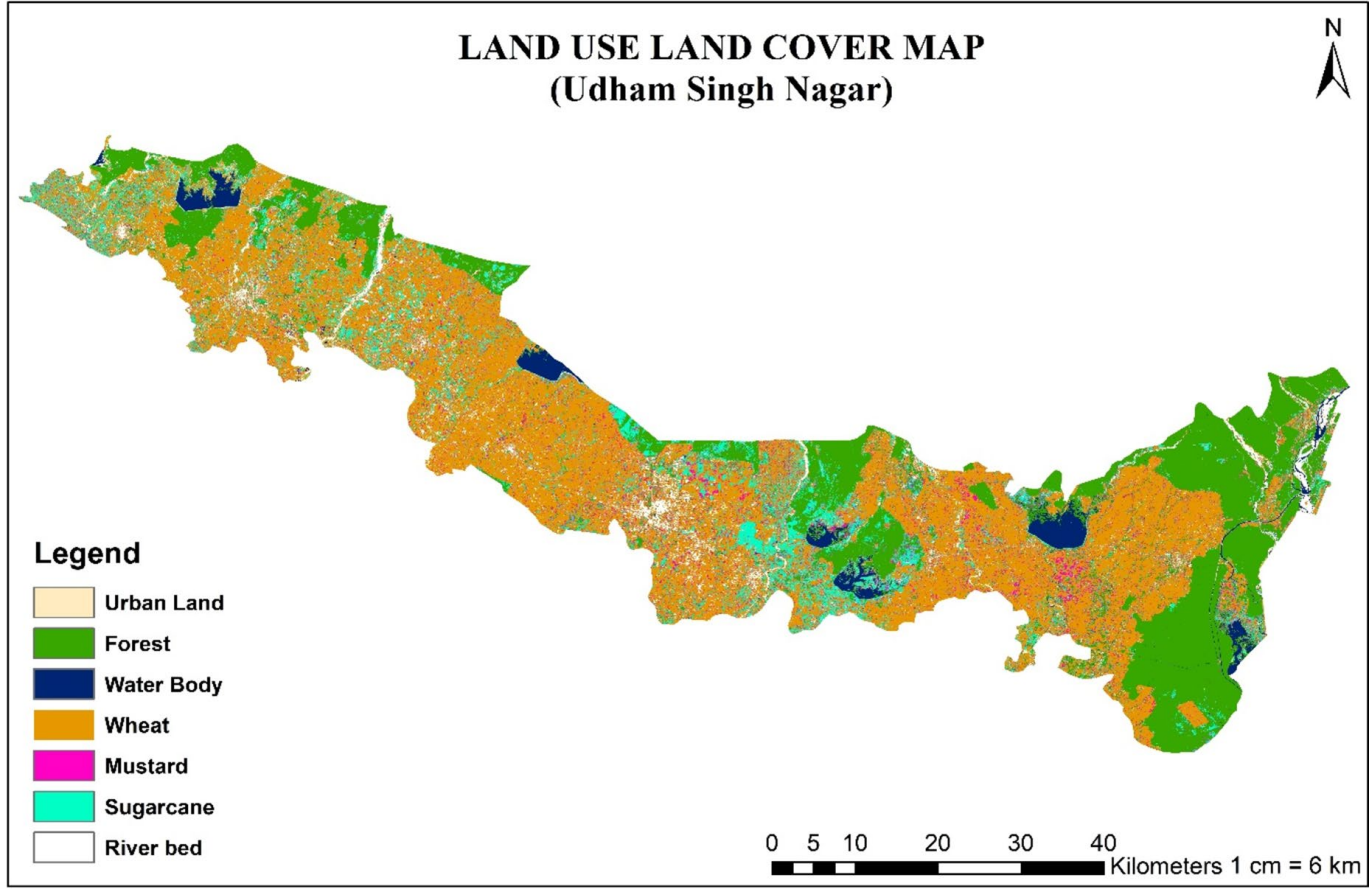
Land use and land cover (LULC) map of the study area for rabi 2022–23.

The focal point of our current investigation lies the Udham Singh Nagar district of Uttarakhand, India, as illustrated in Fig. 1, spanning from longitude 78º45′ E to 80º08′ E and latitude 28º53′ to 29º23′ N. The study area map was created using ArcGIS 10.8.2 software^51^. This locale experiences a mean annual precipitation of 1400 mm, with a striking 80% of this moisture bestowed upon the region during the transformative months of June to September due to courtesy of the South-West monsoon. Rice, wheat, sugarcane, and pulses are the main crop grown in the study area. The land use and land cover map of the study area during rabi season of 2022–23 was prepared based on the ground truth data of rabi season (Fig. 2). The LULC map was created using ArcGIS 10.8.2 software^51^.

The application of the MPT approach for estimating crop evapotranspiration has been reported in various regions worldwide, including the United States, China, and India. However, there is a lack of research on the use of the remote sensing-based approach for estimating crop evapotranspiration in the Udham Singh Nagar district of Uttarakhand. Out of total geographical area of Uttarakhand state, 86% is hilly area and only 14% area is plain area^52^. Due to this topographical difference of the state, only about 14% of the geographical area is well cultivable. Apart from this the state is also having 61% of area under forests (State profile, Government of Uttarakhand). Uttarakhand has total thirteen districts; out of these, only two districts of the state mainly contribute as plain region viz. Haridwar and Udham Singh Nagar. Udham Singh Nagar is selected for this study purposefully since this district is having highest agricultural crop area as compared to other districts of Uttarakhand state (Directorate of economics and statistics). Udham Singh Nagar region lies is the Tarai belt at the foothills of the Shivalik range of Himalayas and about 80% of the crop area is irrigated area (Krishi Vigyan Kendra, Udham Singh Nagar). The higher percentage of the irrigated area also signifies the importance of the evapotranspiration study over this district for better irrigation and water management.

### Remotely sensed data

In this research endeavour, pivotal remotely sensed data crucial for our investigation were sourced from the esteemed United States Geological Survey (USGS) via their website https://earthexplorer.usgs.gov/ accessed on 21st June 2023 for the rabi season of 2022–23. The focus of our scrutiny lies in the multidimensional analysis of multispectral and thermal remote sensing data harnessed from the imagery of Landsat 8 and 9 satellites during the chickpea crop grown in lysimeter (January to April) were used. These satellites, equipped with optical bands boasting a remarkable 30 m resolution and thermal bands at a resolution of 100 m, serve as invaluable tools in our quest for understanding. Leveraging the optical bands, we engage in the calculation of albedo and vegetation indices, while the thermal band becomes instrumental in the precise determination of land surface temperature. The selection of cloud free images process led us to select a total of 9 cloud-free images, spanning from the 17th of December to the 16th of April. This carefully curated dataset forms the foundation for the parametrization of the MPT model, facilitating the estimation of evapotranspiration.

### Meteorological data

Daily agroclimatic parameters, specifically the 2 m surface temperature and surface pressure were meticulously acquired from the ERA 5 repository, a trove of climatic information accessible via the Copernicus EU website, as of the 21st of June 2023. The indispensability of both surface temperature and surface pressure at the time of each satellite passage became apparent, laying the foundation for our subsequent analytical endeavours. It is imperative to note that ERA5, standing as the fifth-generation bastion of reanalysis products, offering a treasure trove of hourly data spanning atmospheric and oceanic domains.

### Lysimeter data

**Fig.3:**
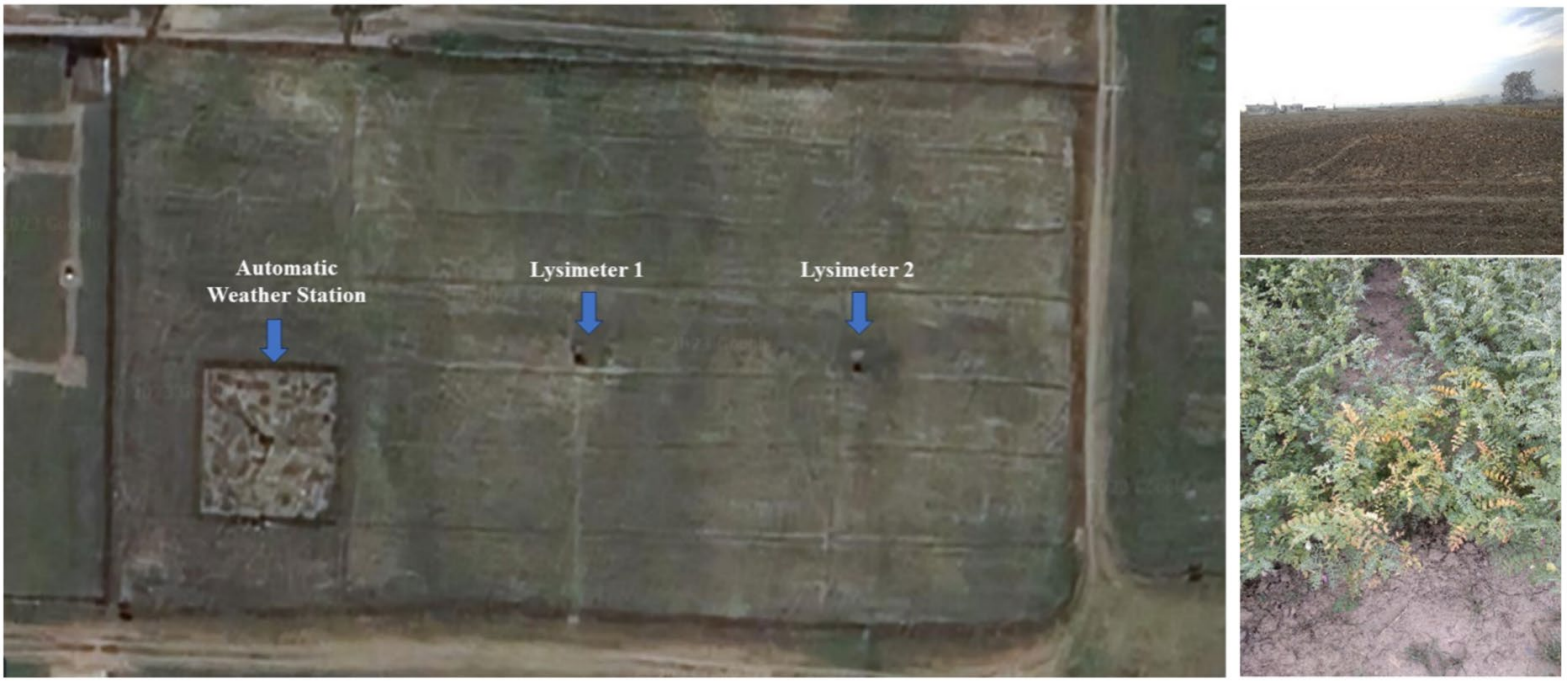
Location map of installed lysimeters, field preparation and grown chickpea crop.

Embarking on a meticulous exploration, a plot-scale lysimeter study unfolded within the experimental domains of the Department of Agrometeorology at GBPUAT, Pantnagar, situated at coordinates 29º 01 ′N 79º 48 ′E, as depicted in Fig. 3. This endeavour unfolded during the Rabi (winter) seasons of 2022–23, showcasing a strategic focus on evaluation of capability of MPT model to predict crop evapotranspiration accurately. The methodology employed a weighing-type lysimeter, serving as a sentinel for daily observations that meticulously documented the nuances of chickpea evapotranspiration. Commencing in the first week of January, the crop was not only cultivated within the confines of the lysimeter tank but also in the proximate field of approximately 4000 m^2^, encapsulating a holistic view of the crop's interaction with its environment. The lysimeter tank, with dimensions measuring 1.33 × 1.33 × 0.9 m^3^, became the controlled arena for this agricultural symphony, while the crop’s manifestation occurred on a platform spanning 120 cm^2^. The crop was harvested at second fortnight of the April month. The lysimetric data is used to verify the results of the MPT model.

## Methodology

### Modified Priestley Taylor model for ET estimation

**Fig.4:**
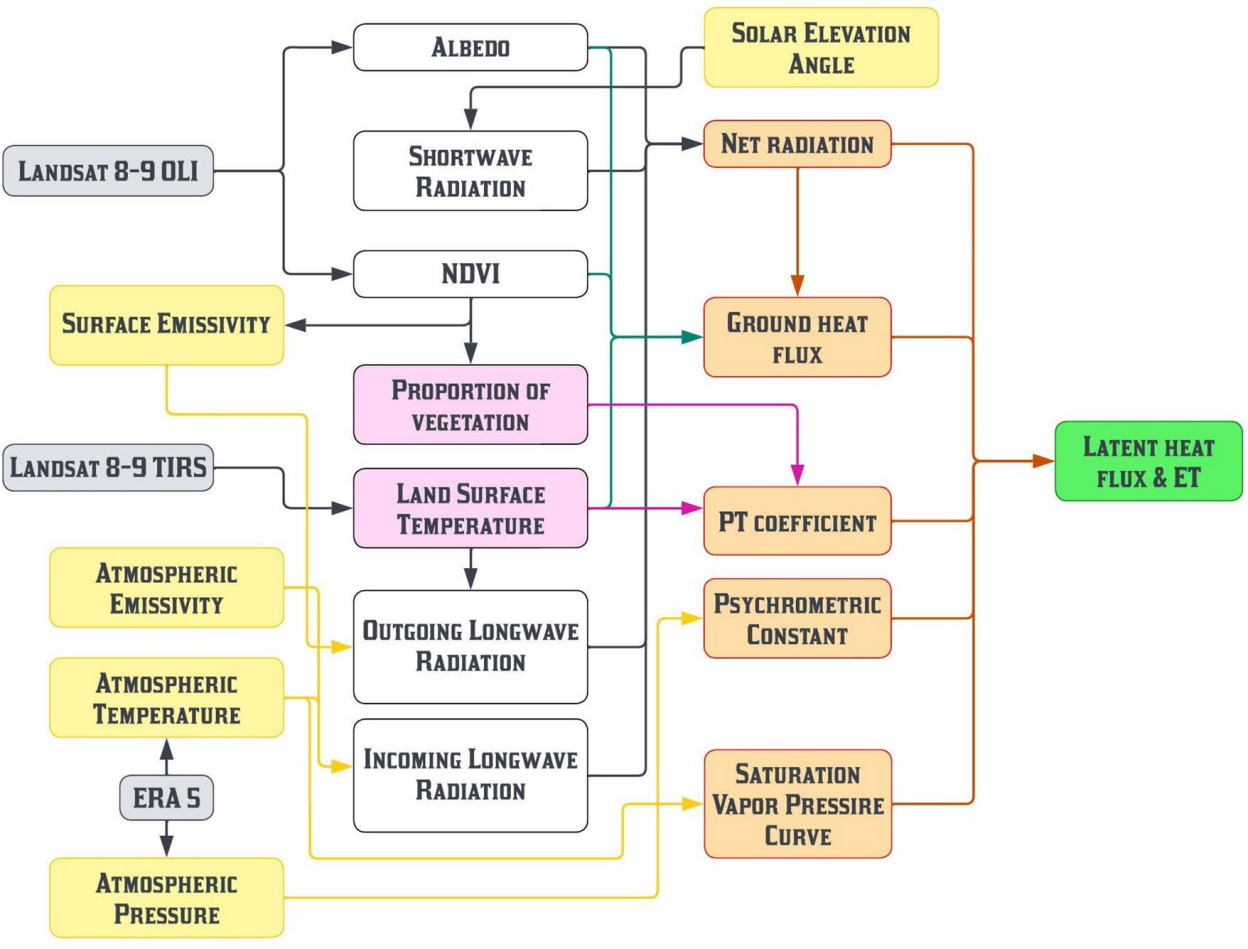
The flowchart of modified Priestley-Taylor model development process.

The reason behind selection of MPT model over several remote sensing-based energy balance method is its simplicity compared to other methods, making it easier to use. An advantage of our novel approach is that it doesn't rely on any ground observations, simplifying the estimation process. However, it's important to note that the MPT model works best in flat areas where elevation changes are minimal, as it mainly uses temperature data, which can vary with elevation. This consideration ensures the method’s suitability for our study area and helps reduce potential errors related to elevation differences. Overall, selecting the MPT model fits our research goals and context, offering a practical and trustworthy approach for ET estimation in our study region. The simplified formula of a Priestley-Taylor method based purely on the remotely sensed data is firstly proposed by Jiang and Islam^28^, can be represented as:

1 $$LE = \phi \left[ {\left( {R_{n} - G} \right)\frac{\Delta }{\Delta + \gamma }} \right]$$

where, ϕ is PT coefficient ranges from 0 to 1.26, R_n_ is net radiation, G is ground heat flux, γ is psychrometric constant and Δ is saturated vapor pressure curve. All the four entities (R_n_, G, ϕ and Δ) can be derived independently majorly using remotely sensed data^28,53^. The Fig. 4 shows the flowchart of all the data required and process followed to develop the MPT model.

The findings of this research offer valuable insights into the spatiotemporal variations of evapotranspiration, which are crucial for effective water resource management and agricultural planning in the area. Importantly, this study also contributes uniquely by demonstrating the applicability of the MPT model in regions where ground-based weather data is limited or unavailable. By relying on reanalysis products such as temperature, rather than ground-based observed data, it showcases the model's versatility and robustness across diverse geographical areas. This aspect underscores the practical utility of this modified approach in regions where traditional weather stations may be sparse or non-existent, thereby expanding the scope of evapotranspiration estimation to previously underserved areas.

### Criteria for selecting specific Landsat images of the MPT model

In our study, we meticulously selected Landsat imagery to ensure high-quality data for analysis. The Landsat data utilized in this research were obtained from the United States Geological Survey (USGS) Earth Explorer database. We primarily focused on Landsat 8 and Landsat 9 imagery due to their superior spatial resolution and spectral characteristics. The specific Landsat data parameters are detailed in Table 3, including the collection category, number, WRS path, WRS row, sensor identifier and datum, which are crucial for accurately identifying and accessing the desired imagery.

**Table 3:** Specific Landsat data parameters.

Information head	Details
Collection category	T1
Collection number	2
WRS path	145
WRS row	040
Sensor identifier	OLI_TIRS
Datum	WGS84

To ensure the reliability of our analysis, we established stringent criteria for selecting Landsat images, with a primary focus on minimizing cloud cover. Table 4 provides detailed information on the Landsat images used in our study, including the acquisition date, Landsat version (8 or 9) and corresponding cloud cover percentage. Notably, we prioritized images with minimal cloud cover to facilitate clear observation of the study area. However, rather than adhering to a fixed threshold of cloud cover percentage, we assessed each image individually to ensure a cloud-free view of the study area that’s why higher cloud cover percentage can be seen on the images of 17th December 2022 and 26th January 2023 (clouds are on other parts of image not above the study area). This approach allowed us to maintain consistency and accuracy across our Landsat image selection process. In summary, our methodology involved meticulous selection of Landsat imagery based on stringent criteria, with a focus on minimizing cloud cover to ensure clarity and accuracy in our analysis.

**Table 4:** Detailed information on the Landsat images used.

Date	Landsat	Cloud cover
17-Dec-22	9	9.04
18-Jan-23	9	0.00
26-Jan-23	8	23.79
11-Feb-23	8	0.84
19-Feb-23	9	1.34
15-Mar-23	8	0.39
23-Mar-23	9	0.22
8-Apr-23	9	0.39
16-Apr-23	8	0.06

### Estimation of surface energy Flux using Landsat Data

#### Net radiation

The net radiation portion mainly consists of two types of radiation viz. longwave radiation and shortwave radiation. The formula provided by Allen et al.^16^ was used to compute the Net radiation:

2 $${R}_{n}=\left(1-\alpha \right){ R}_{s\downarrow }+{R}_{L\downarrow }-{R}_{L\uparrow }-\left(1-{\varepsilon }_{o}\right) {R}_{L\downarrow }$$

where, $R_{n}$ is net radiation, $\alpha$ is albedo, ${R}_{s}$ is shortwave radiation, ${R}_{L}$ is longwave radiation, ${\varepsilon }_{0}$ is surface emissivity, $\downarrow$ is denoting incoming radiation and $\uparrow$ is denoting outgoing radiation. Tasumi et al.^54^ developed an algorithm for calculating at-surface broad-band albedo. it can be calculated as follows:

3 $$\alpha = 0.254 \times \rho_{2} + 0.149 \times \rho_{3} + 0.147 \times \rho_{4} + 0.311 \times \rho_{5} + 0.103 \times \rho_{6} + 0.036 \times \rho_{7}$$

where, $\uprho$ represents reflectance values of Landsat bands 2,3,4,5,6 and 7. ${\text{R}}_{\text{s}\downarrow }$ is calculated, assuming clear sky conditions as (Waters et al.^55^):

4 $$R_{s \downarrow } = G_{sc} \times \cos \theta \times d_{r} \times \tau_{sw}$$

where, $G_{sc}$ is the solar constant (1367 W/m^2^), $cos \theta$ is the cosine of the solar incidence angle, ${\text{d}}_{\text{r}}$ is the inverse squared relative sun-earth distance, and ${\uptau }_{\text{sw}}$ is the broad band atmospheric transmittivity. The cosine of the solar incidence angle is simply derived from the sun elevation angle provided in the metadata file of the Landsat images:

5 $$\text{cos}\theta =\text{cos}\left(\frac{\pi }{2}-\varphi \right)$$

where, $\varphi$ is sun elevation angle in radians. Now, ${\tau }_{sw}$ can be calculated with two separate components for beam and diffused radiation (Allen^56^):

6 $${\uptau }_{\text{sw}}= {\uptau }_{\text{B}}+{\uptau }_{\text{D}}$$

where, ${\uptau }_{\text{B}}$ is the transmittivity index for direct beam radiation and ${\uptau }_{\text{D}}$ is the transmittivity index for diffuse radiation. The calculation of ${\uptau }_{\text{B}}$ can be done with following formula given by (Allen^56^):

7 $$\tau_{B} = 0.98\exp \left[ {\frac{{ - 0.00146P_{air} }}{{K_{t} \cos \theta }} - 0.075\left( {\frac{W}{\cos \theta }} \right)^{0.4} } \right]$$

where, ${\text{P}}_{\text{air}}$ is air pressure (kPa) obtained through ERA5 data, ${\text{K}}_{\text{t}}$ is a unitless “clearness” coefficient 0<${\text{K}}_{\text{t}}$  <  = 1.0 where ${\text{K}}_{\text{t}}$ = 1.0 for clean air and ${\text{K}}_{\text{t}}$ = 0.5 for extremely turbid, dusty, or polluted air (Usually ${\text{K}}_{\text{t}}$ is set equal to 1), θ is the solar incidence angle and W is precipitable water in the atmosphere (mm). Precipitable water is calculated as following (Garrison and Adler^57^):

8 $$W = 0.14 e_{a} P_{air} + 2.1$$

where, ${\text{e}}_{\text{a}}$ is near surface vapour pressure (kPa) calculated through ERA5 temperature data. After calculating ${\uptau }_{\text{B}}$, the ${\uptau }_{\text{D}}$ is estimated from ${\uptau }_{\text{B}}$ itself as (Allen^56^):

9 $${\uptau }_{\text{D}}=0.35-0.36{\uptau }_{\text{B}}\text{ for }{\uptau }_{\text{B}} \ge 0.15$$

10 $${\uptau }_{\text{D}}=0.18+0.82 {\uptau }_{\text{B}}\text{ for }{\uptau }_{\text{B}} <0.15$$

The ${\text{d}}_{\text{r}}$ was computed using the following equation by Duffie and Beckman (1980), which was also used by Allen et al.^16^:

11 $${\text{d}}_{\text{r}}=1+0.033\text{cos}\left(\text{DOY}\frac{2\uppi }{365}\right)$$

where; DOY is the sequential day of the year. Values of ${\text{d}}_{\text{r}}$ ranges from 0.97 to 1.03 and are dimensionless^55^. The incoming longwave radiation is the downward thermal radiation flux, initiated by the atmosphere (W/m^2^). It can be computed using the Stefan-Boltzmann equation:

12 $$R_{L \downarrow } = \varepsilon_{a} \times \sigma \times T_{a}^{4}$$

where; ${\upvarepsilon }_{\text{a}}$ is the effective atmospheric emissivity (dimensionless), $\upsigma$ is the Stefan-Boltzmann constant ($5.67\times {10}^{-8}\text{ W}/{\text{m}}^{2}/{\text{K}}^{4}$) and ${\text{T}}_{\text{a}}$ is the near surface air temperature (K) which can be taken from ERA5 product. To calculate ${\upvarepsilon }_{\text{a}}$, empirical equation provided by Bastiaanssen^58^ can be used by using coefficients developed by Allen (2000)^17^:

13 $$\varepsilon_{a} = 0.85 \times \left( { - \ln \tau_{sw} } \right)^{0.09}$$

The outgoing longwave radiation (${\text{R}}_{\text{L}\uparrow }$) is calculated using the Stefan-Boltzmann equation:

14 $${R}_{L\uparrow }={\varepsilon }_{o}\times \sigma \times {{T}_{s}}^{4}$$

where; $\upsigma$ is the Stefan-Boltzmann constant ($5.67\times {10}^{-8}\text{ W}/{\text{m}}^{2}/{\text{K}}^{4}$) and ${\text{T}}_{\text{s}}$ is the land surface temperature (K), which can be calculated by the single-channel algorithm proposed by Jiménez‐Muñoz and Sobrino^59^ and ${\upvarepsilon }_{\text{o}}$ is the broad-band surface emissivity (dimensionless). Van De Griend and Owe^60^ has given a formula to calculate broad-band surface emissivity:

15 $$\varepsilon_{o} = 1.009 + 0.047\ln \left( {NDVI} \right) for NDVI > 0$$

where; emissivity is assumed to be zero if NDVI > 0 (for example, for water).

#### Soil heat flux (G)

The ratio of the ground heat flux and net radiation using the empirical equation provided by Bastiaanssen^58^, representing values near mid-day:

16 $$\frac{G}{{R}_{n}}=\frac{{T}_{s}}{\alpha }\left(\left(0.0038 \alpha +0.0074 {\alpha }^{2}\right)\left(1-0.98 {NDVI}^{4}\right)\right)$$

#### Calculation of Priestley-Taylor (PT) coefficient ($\upphi$)

The vegetation index which will be calculated for this purpose will be fraction of vegetation (${\text{F}}_{\text{r}}$) for each pixel proposed by Carlson and Ripley^61^:

17 $${\text{F}}_{\text{r}}={\left(\frac{\text{NDVI}-{\text{NDVI}}_{\text{min}}}{{\text{NDVI}}_{\text{max}}-{\text{NDVI}}_{\text{min}}}\right)}^{2}$$

**Fig.5:**
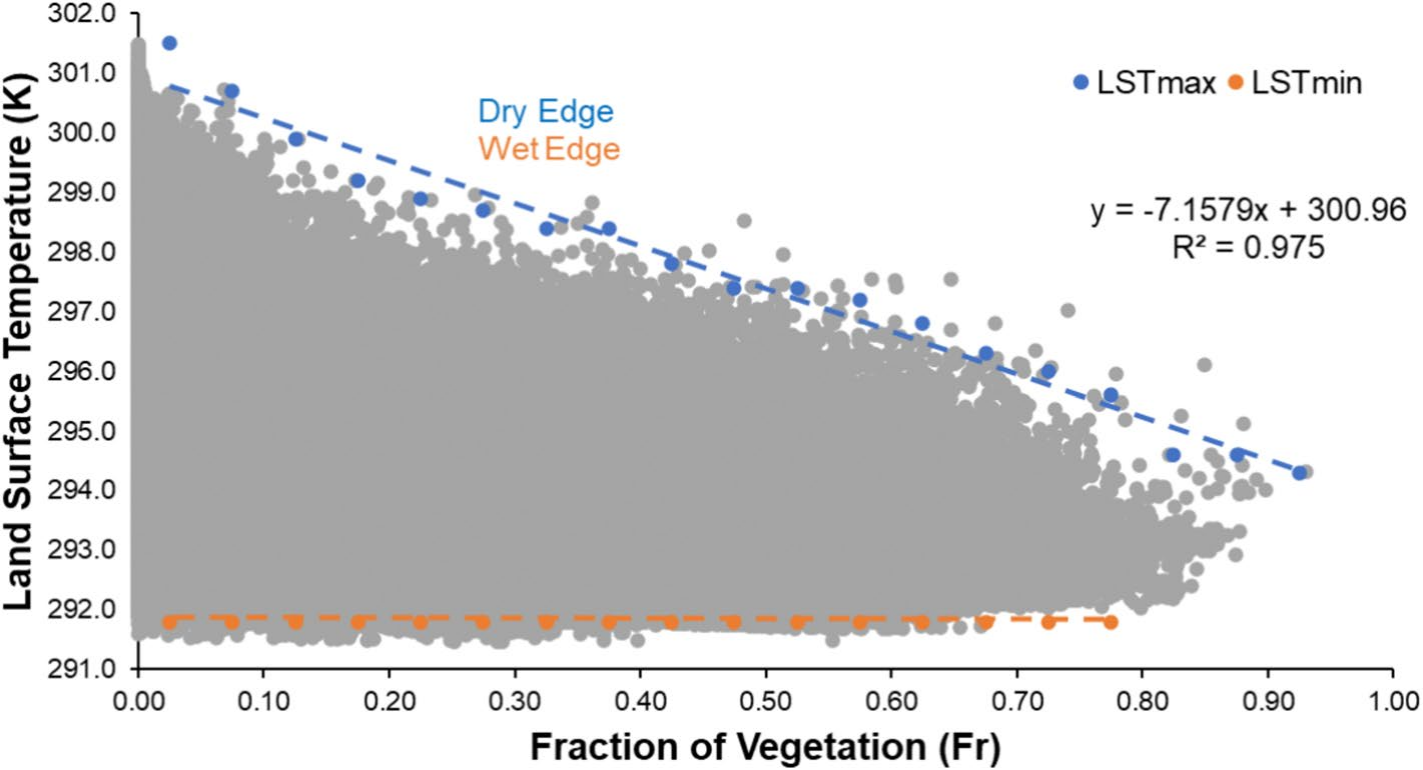
Scatter plot between surface temperature and fraction of vegetation.

In this study, the direct incorporation of irrigation practices was not explicitly modelled. However, the influence of water availability, including the effects of irrigation, is indirectly embedded in the Modified Priestley-Taylor (MPT) model through the use of NDVI (Normalized Difference Vegetation Index) and temperature data. The estimated ET in this study is largely driven by these two parameters. Specifically, higher NDVI values typically indicate good water availability, which may result from sufficient irrigation or natural precipitation, while lower NDVI values can reflect water stress conditions. In this way, the MPT model inherently assumes water sufficiency in areas with higher NDVI values, indirectly incorporating the impact of irrigation. This approach aligns with the underlying assumptions of the MPT model, which is designed to estimate potential ET under conditions of sufficient water supply. After calculation of the ${\text{T}}_{\text{s}}$ and ${\text{F}}_{\text{r}}$ for each pixel, a scatter plot was made against each other for the calculation of dry (upper decreasing line) and wet edge (lower nearly horizontal line), as shown in Fig. 5.

After having the determines dry and wet edge, the value of $\upphi$ corresponding to the dried bare soil is set to 0 and the value of $\upphi$ at maximum vegetation and lowest temperature is set to 1.26. Then two-step linear interpolation is used to get the value of $\upphi$ for each pixel. For every pixel, it can be calculated as^29^:

18 $$\phi = \frac{{T_{\max ,i} - T_{s,i} }}{{T_{\max ,i} - T_{\min ,i} }}\left( {\phi_{\max ,i} - \phi_{\min ,i} } \right) + \phi_{\min ,i}$$

In which; ${\upphi }_{\text{max},\text{ i}}={\upphi }_{\text{max}}=1.26$ and ${\upphi }_{\text{min},\text{ i}}=1.26 {\text{F}}_{\text{r}}$. The psychrometric constant relates the partial pressure of water in the air to the air temperature. The psychrometric constant ($\upgamma$) is given by formula (Allen et al.^16^):

19 $$\gamma = \frac{{C_{p} P}}{\varepsilon \lambda }$$

where, ${\text{C}}_{\text{p}}$ is specific heat at constant pressure (1.013 $\times$ 10^–3^ MJ kg^-1^ ℃^−1^), P is atmospheric pressure, $\upvarepsilon$ is ratio of molecular weight of water vapour/dry air (0.622) and $\uplambda$ is latent heat of vaporization (2.45 MJ kg^-1^). The slope of saturation vapour pressure curve ($\Delta$) at a given temperature is given by Allen et al.^16^:

20 $$\Delta = \frac{4098 \left[0.6108\text{exp}\left(\frac{17.27\text{ T}}{\text{T}+237.3}\right)\right]}{{(\text{T}+237.3)}^{2}}$$

The calculated value of LE was then converted into instantaneous ET (ET_i_) by using the formula:

21 $$ET_{i} = 3600 \times \frac{LE}{\lambda }$$

The upscaling of instantaneous ET to daily ET was done by many methods, developed by many researchers, but MEF (modified evaporative fraction) is used widely. EF (evaporative fraction) method usually underestimates the value of daily ET^62^. Thus, the value of daily ET was calculated with following expression:

22 $$ET_{d} = \alpha \times EF_{i} \times \left( {R_{n} - G} \right)_{d}$$

where, $\alpha$ is a modified coefficient. For $\alpha$ many researchers by default take the value of 1.1^63–66^.

### Model evaluation

For testing the developed model with respect to the lysimetric data five parameters viz. coefficient of determination (R^2^), root mean square error (RMSE), Nash–Sutcliffe efficiency parameter (NSME), agreement index (d) and mean biased error (MBE) were used is this study. The R^2^, RMSE, NSME, d and MBE are estimated as^67,68^:

23 $$R^{2} = \left( {\frac{{\frac{1}{n}\mathop \sum \nolimits_{i = 1}^{n} \left( {y_{i} - \overline{y}_{i} } \right)\left( {\hat{y}_{i} - \overline{\hat{y}}_{i} } \right)}}{{\sigma_{y} \sigma_{{\hat{y}}} }}} \right)^{2}$$

24 $$RMSE=\sqrt{\frac{{\sum }_{i=1}^{n}{\left({y}_{i}-{\widehat{y}}_{i}\right)}^{2}}{n}}$$

25 $$d=1-\left(\frac{{\sum }_{i=1}^{n}{\left({y}_{i}-\widehat{{y}_{i}}\right)}^{2}}{{ {\sum }_{i=1}^{n}\left(\left|\widehat{{y}_{i}}-\overline{{y }_{i}}\right|+\left|{y}_{i}-\overline{{y }_{i}}\right|\right)}^{2}}\right)$$

$$NSME=1- \frac{{\sum }_{i=1}^{n}{\left({y}_{i}-{\widehat{y}}_{i}\right)}^{2}}{{\sum }_{i=1}^{n}{\left({y}_{i}-\overline{{y }_{i}}\right)}^{2}}$$

$$MBE=\frac{1}{n}{\sum }_{i=1}^{n}\left({y}_{i}-{\widehat{y}}_{i}\right)$$

Here, ${y}_{i}$ is the observed value, ${\widehat{y}}_{i}$ is the predicted value, $\overline{{y }_{i}}$ is the mean of observed values, ${\sigma }_{y}$ and ${\sigma }_{\widehat{y}}$ is the standard deviation of actual and predicted values respectively and n is number of observations.

The value of R^2^ can range from 0 to 1 and value 1 illustrates a strong linear relationship. The lower value of RMSE shows better model performance and higher value shows poor model performance^69,70^. The value of NSME ranges from -∞ to 1. The NSME value close to 1 shows better model efficiency, the value of 0 indicates model accuracy close to accuracy of the calculated mean of observed data and negative value shows insufficiency of the model. The value of d ranges between 0 to 1. The 1 value shows the perfect match between observed and predicted values while 0 value shows no match between them^2,71,72^. The value of MBE indicates the average bias in the prediction. A positive value of MBE indicates overestimation and negative value of MBE indicates underestimation from the datasets^4,73^.

## Results

### Validation of daily ET through lysimetric data

In the meticulous effort to validate the daily evapotranspiration (ET) estimates generated by the Modified Priestley-Taylor (MPT) model, a thorough comparison was conducted using lysimetric daily ET data specifically for chickpea cultivation. With chickpea planting initiating in January, a total of 8 daily ET values were collected and analyzed to compare against their lysimetric counterparts. The outcomes of this comparative analysis revealed a significant agreement between the daily ET values estimated by the MPT model and the lysimetric data, supported by a strong R2 value of 0.71. Further metrics of model performance bolstered this assertion, with the Nash–Sutcliffe Model Efficiency (NSME) reaching a praiseworthy 0.66, indicative of robust model performance. Additionally, the Agreement Index (d) soared to an impressive 0.91, underscoring the MPT model's excellence in capturing the nuances of ET dynamics. The Mean Bias Error (MBE) value, hovering close to zero at 0.04 mm day−^1^, further reinforced the model's aptitude for accurate agreement.

**Fig.6:**
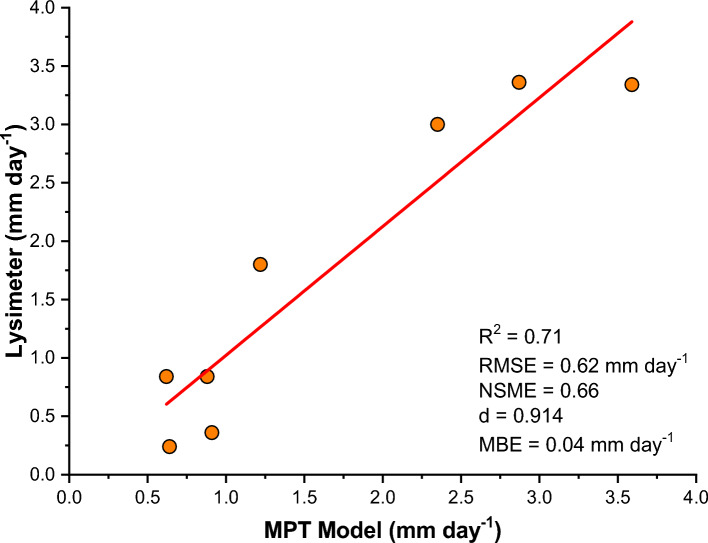
Scatter plot between measured and estimated values of ET.

These findings collectively advocate for the efficacy of the MPT model in the precise estimation of ET over the canvas of Udham Singh Nagar district. The resonance between the measured and estimated ET values is vividly depicted in Fig. 6, encapsulating a visual testament to the high degree of correlation between the two datasets. In essence, the MPT model emerges as a potent tool, demonstrating its capacity for accurate ET retrieval in the dynamic agricultural landscape of Udham Singh Nagar district, Uttarakhand, India.

### Validation of daily ET through EEFlux data

A reference for general equations for EEFlux, based on those of METRIC is available at: http://www.intechopen.com/books/evapotranspiration-remote-sensing-and-modeling/operational-remote-sensing-of-et-and-challenges which is an Intech book chapter compiled by Dr. Ayse Kilic (Irmak) of the Univ. Nebraska-Lincoln and associates at the University of Idaho and Desert Research Institute in 2012. An original reference for METRIC is Allen et al.^17^ published in the ASCE J. Irrigation and Drainage Engineering. The outcomes of this comparative investigation revealed a commendable agreement between the daily ET values calculated from the MPT model and the lysimetric data, as evidenced by a strong R^2^ value of 0.83. Additional metrics assessing model performance supported this finding, with the Nash Sutcliffe model efficiency (NSME) reaching a noteworthy -0.23, indicative of robust model performance. Moreover, the Agreement Index (d) surged to an impressive 0.81, highlighting the exceptional ability of the MPT model to capture the intricacies of ET dynamics. The mean bias error (MBE) and root mean squared error (RMSE) value, quite more as 1.02 and 1.19 mm day−^1^ respectively, further emphasized the model's accuracy in agreement.

**Fig.7:**
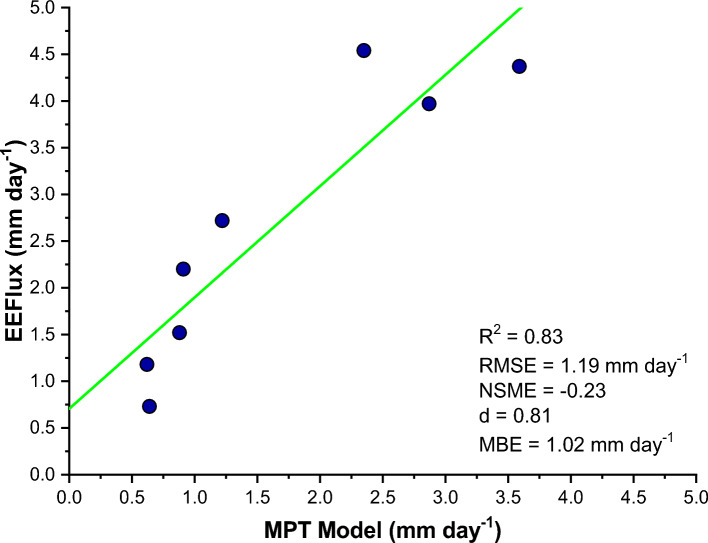
Scatter plot between model and EEFlux values of ET.

These results strongly support the effectiveness of the MPT model in accurately estimating ET across the landscape of Udham Singh Nagar district. The clear alignment between observed and estimated ET values is vividly illustrated in Fig. 7, serving as a visual confirmation of the significant correlation between the datasets. Essentially, the MPT model stands out as a powerful tool, showcasing its capability for precise ET retrieval within the dynamic agricultural setting of Udham Singh Nagar district, Uttarakhand, India.

### Spatiotemporal variation of energy fluxes

**Fig.8:**
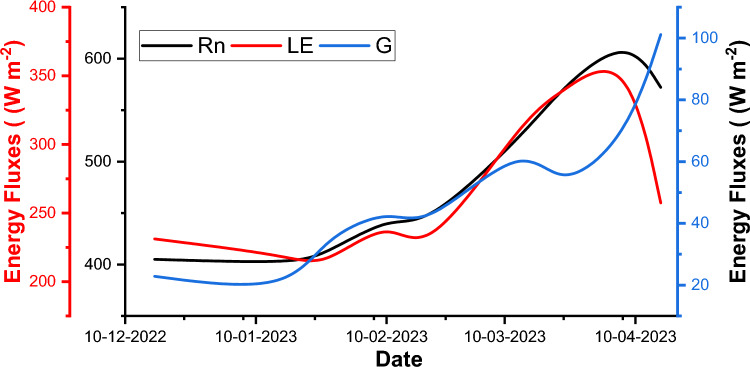
Temporal variation of mean values of energy flux over study region.

In the intricate choreography of parameterizing the Modified Priestley-Taylor model, the initial ballet unfolds with the meticulous quantification of net radiation (R_n_). The value of R_n_ was obtained after getting the values of incoming short, incoming and outgoing long wave radiation^74,75^. The temporal variation in the values of energy fluxes viz*.* R_n_, G and LE over the canvas of Udham Singh Nagar is artfully presented in Table 5 and visualised in Fig. 8. The mean values of different energy fluxes range as mentioned here: R_n_ from 401.14 Wm^−2^ (January) to 623.67 Wm^−2^ (April), G from 17.25 Wm^−2^ (January) to 101.17 Wm^−2^ (April) and LE from 209.99 Wm^−2^ (January) to 372.11 Wm^−2^ (April). The low temperature, fog and haze are the main causes for the lower values of Rn during the winter months^76^. The lower values of energy fluxes during the December month are associated with the lower temperatures and foggy conditions over the study region.

**Table 5:** Spatiotemporal mean values of energy fluxes.

Date	R_n_	G	G/R_n_	LE
17-Dec-22	405.08	22.86	0.06	231.19
18-Jan-23	401.14	17.25	0.04	218.87
26-Jan-23	408.71	35.39	0.09	209.99
11-Feb-23	447.83	44.99	0.10	248.31
19-Feb-23	434.95	37.66	0.09	214.23
15-Mar-23	528.32	67.82	0.13	321.32
23-Mar-23	569.72	49.73	0.09	337.14
08-Apr-23	623.67	67.57	0.11	372.11
16-Apr-23	572.10	101.17	0.18	257.41

*R*_*n*_ net radiation, G ground heat flux, *LE* latent heat flux.

In our quest for comprehensive insights, the values of G/R_n_ ratio were calculated for different time periods. G/R_n_ ratio values range from 0.04 (January) to 0.18 (April) suggesting higher portion of net radiation is used for ground heat flux during the April month as compared to other months. The G/R_n_ ratio varies from 0.04 to 0.15 for crops^55^. However, G/R_n_ ratio values varies from 0.2 to 0.4 for bare soil and 0.5 value indicates clear water or snow. Kustas and Daughtry^77^ also studied G/R_n_ ratio and found 0.15 value for crop field. Hence, G/R_n_ ratio of this study is in line with the previous studies.

### Spatiotemporal variation of daily ET and NDVI

The daily evapotranspiration (ET) values for the study area were computed based on the theoretical framework elucidated in the methodology section, with Table 6 encapsulating the comprehensive dataset of these daily ET values and their fluctuations within the study area. A detailed analysis of the ET variations across Udham Singh Nagar district unveiled a distinct pattern: December (1.33 mm/day) < January (1.57 mm/day) < February (1.7 mm/day) < March (2.99 mm/day) < April (3.2 mm/day).

**Table 6:** Daily average evapotranspiration in Udham Singh Nagar district.

Date	Max. (mm/day)	Daily Mean ET (mm/day)	SD
17-Dec-23	2.10	1.33	0.29
18-Jan-23	2.46	1.50	0.33
26-Jan-23	2.59	1.64	0.32
11-Feb-23	3.22	1.80	0.48
19-Feb-23	2.99	1.60	0.33
15-Mar-23	4.58	2.73	0.78
23-Mar-23	5.74	3.25	0.60
08-Apr-23	9.42	3.68	1.14
16-Apr-23	6.61	2.71	0.92

*ET* evapotranspiration, *Max* maximum, *SD* standard deviation.

**Fig.9:**
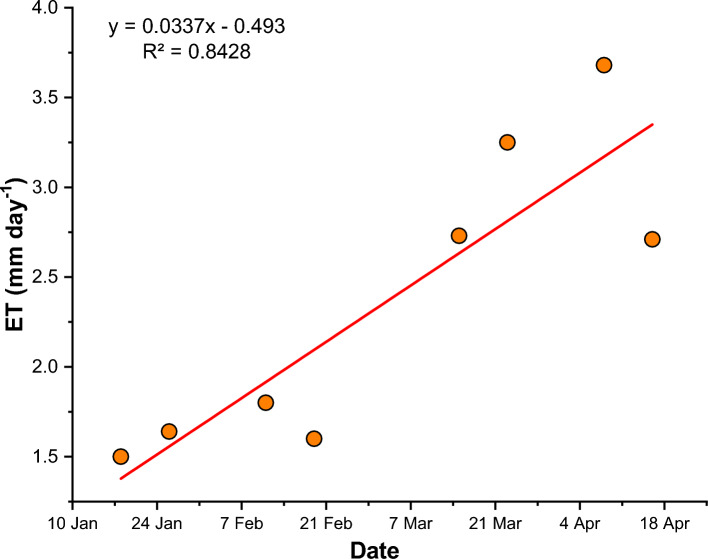
Scatter plot of daily ET values during different months.

This progressive cadence is visually represented in Fig. 9, illustrating the evolving relationship between daily ET values and the temporal trajectory (months). The robust correlation coefficient, with an R^2^ value of 0.84, substantiates the upward trend of daily ET values with the passage of months. This temporal evolution finds its rationale in the shifting local climatic dynamics across different seasons. December, characterized as one of the coldest months, experiences limited thermal conditions that suppress the ET process. Subsequently, as temperatures gradually ascend, the ET process commences a gradual intensification, towards the month of April. Therefore, Udham Singh Nagar shows highest ET during April month due to optimal hydrothermal condition. In summary, the local weather conditions which influence the hydrothermal condition is the main factor determining the rate of daily ET in the study region.

**Fig.10:**
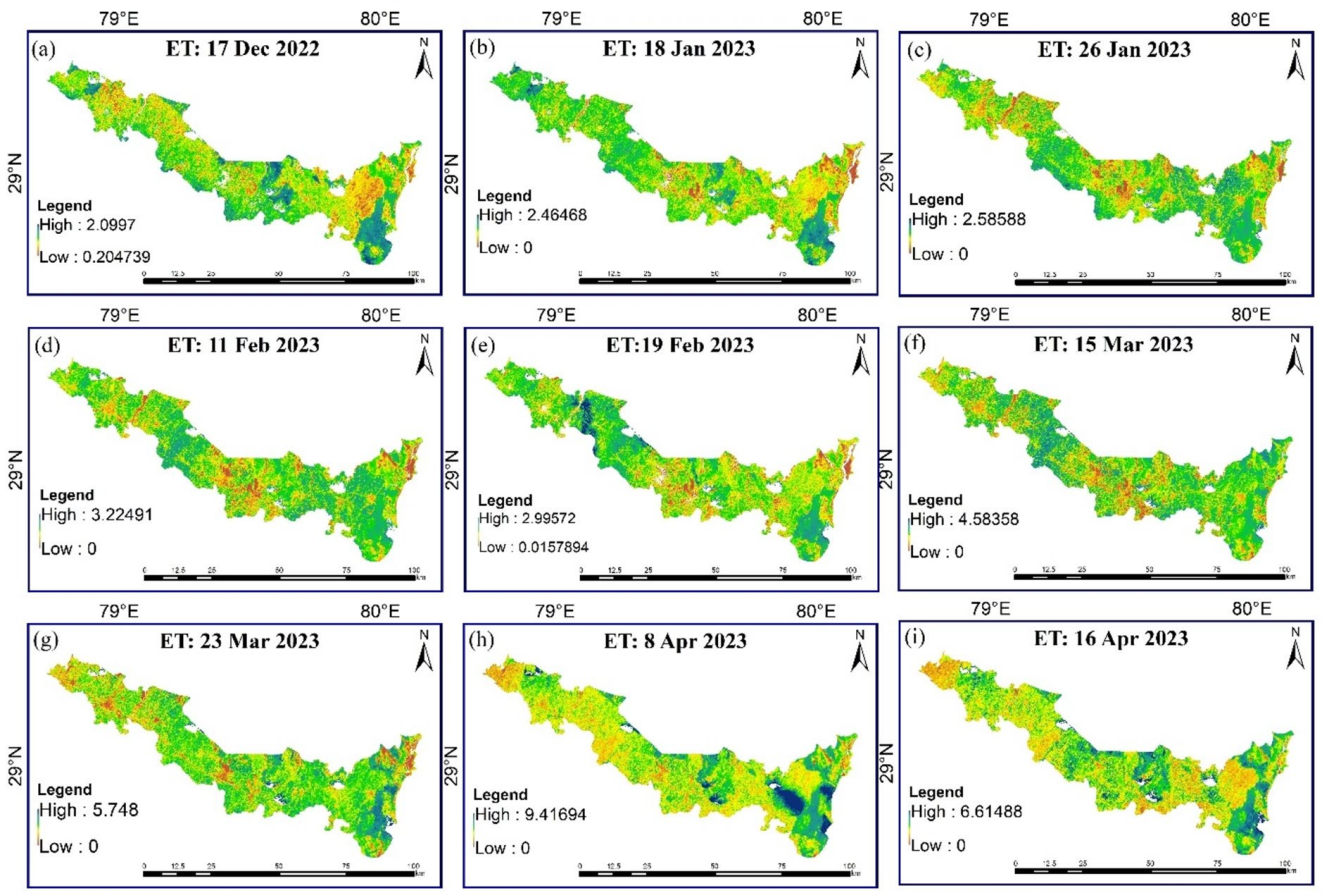
Spatiotemporal distribution of daily ET over Udham Singh Nagar.

Figure 10, 11 shows the spatial and temporal variation of daily ET and NDVI over the study region, respectively, was created using ArcGIS 10.8.2 software^51^. Notably, the southeastern sector emerges as an ET hotspot, consistently exhibiting higher rates compared to other locales throughout the observation period. This heightened ET phenomenon is notably concentrated in areas adorned with dense forest and woodland cover, which is supported by higher NDVI values over that area and the LULC map of the study area (Fig. 2). Conversely, the central expanse of the study region showcases a distinctive temporal pattern, with ET levels soaring up until March before undergoing a sharp reduction. This intriguing fluctuation aligns with the agricultural rhythm of the region, where crop lands dominate the landscape as depicted in LULC map of the study area (Fig. 2). Consequently, the diminishing ET and NDVI values in the later months align with the natural progression of the crop cycle. During January *rabi* crops can be found in these areas at their peak vegetative stage which comes to maturity in March and April month. Hence, the value of ET and NDVI also decreases with the maturity of the *rabi* crops. Contrastingly, the northernmost and lower regions, characterized by sparse vegetation and urban land conditions, exhibit comparatively lower ET and NDVI values.

**Fig.11:**
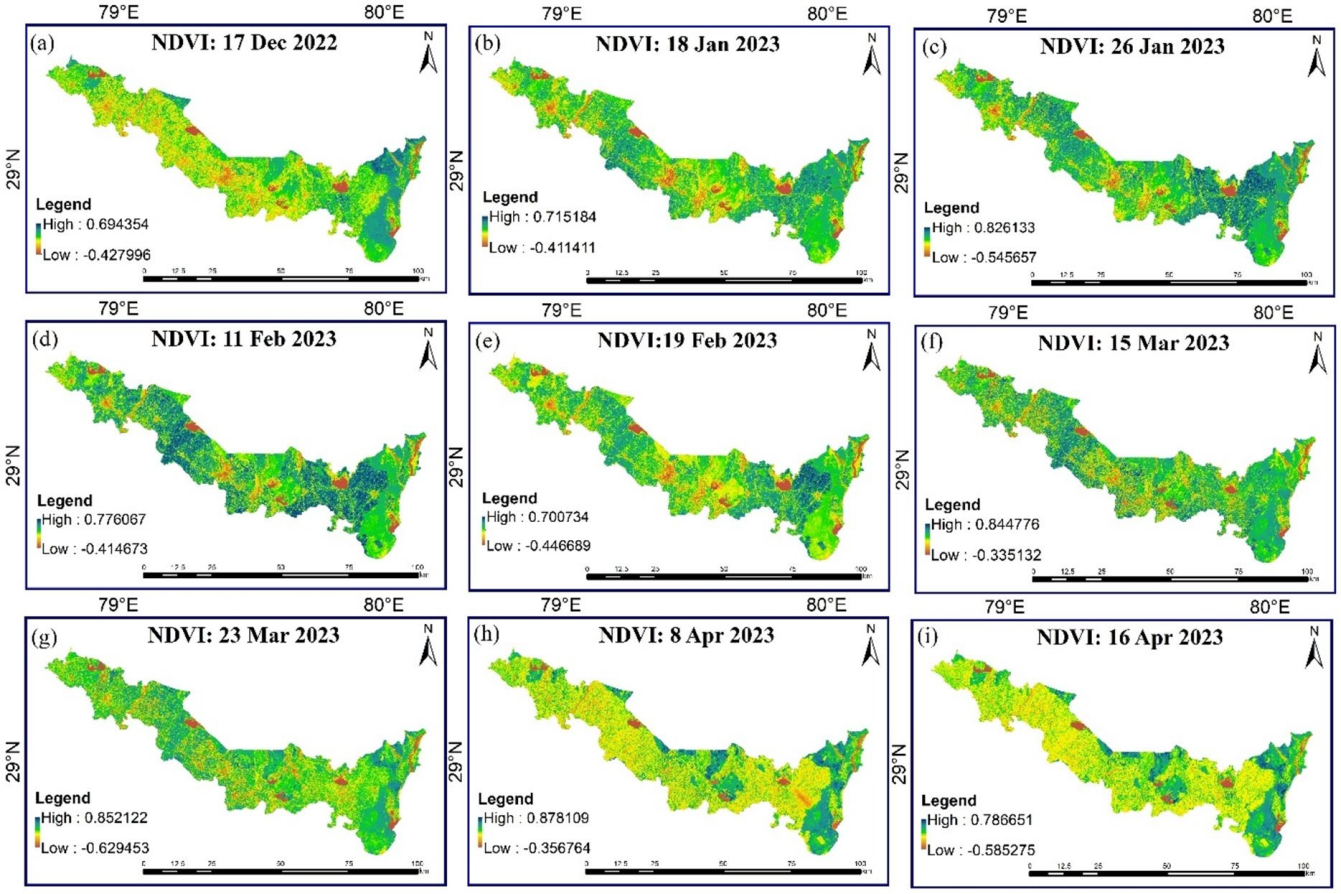
Spatiotemporal variation of NDVI over Udham Singh Nagar.

In interpreting the results of the daily evapotranspiration (ET) variation over the study area, considered several factors and processes that influence ET dynamics. Our analysis revealed a distinct seasonal pattern, with ET rates gradually increasing from December to April. This progression can be attributed to shifting local climatic dynamics, with December experiencing limited thermal conditions due to colder temperatures, which suppress the ET process. As temperatures gradually rise, particularly towards April, the ET process intensifies due to optimal hydrothermal conditions. Factors such as land use and land cover, as depicted in the Land Use and Land Cover (LULC) map of the study area, also play a significant role in influencing ET patterns. For instance, areas with dense forest and woodland cover exhibited higher ET rates, while regions dominated by sparse vegetation and urban land conditions displayed comparatively lower ET values. Additionally, agricultural activities, such as the cultivation of rabi crops, contribute to fluctuations in ET levels, with peak vegetative stages in January gradually transitioning to maturity in March and April. While our study focused on analyzing the spatiotemporal variation of daily ET using Landsat imagery data, we acknowledge the importance of comparing and contrasting our results with previous studies and literature. Unfortunately, due to the specific scope and focus of our research, direct comparisons with previous studies were limited. However, our findings align with established principles of ET dynamics, emphasizing the influence of climatic factors, land use, and land cover on ET patterns. Future research efforts could further explore the nuances of ET variation and validate our findings against existing literature to enhance the robustness of our conclusions.

## Discussion

In interpreting the results of the daily evapotranspiration (ET) variation over the study area, we considered several factors and processes that influence ET dynamics. Our analysis revealed a distinct seasonal pattern, with ET rates gradually increasing from December to April. This progression can be attributed to shifting local climatic dynamics, with December experiencing limited thermal conditions due to colder temperatures, which suppress the ET process. As temperatures gradually rise, particularly towards April, the ET process intensifies due to optimal hydrothermal conditions. Factors such as land use and land cover, as depicted in the Land Use and Land Cover (LULC) map of the study area, also play a significant role in influencing ET patterns. For instance, areas with dense forest and woodland cover exhibited higher ET rates, while regions dominated by sparse vegetation and urban land conditions displayed comparatively lower ET values.

Additionally, agricultural activities, such as the cultivation of rabi crops, contribute to fluctuations in ET levels, with peak vegetative stages in January gradually transitioning to maturity in March and April. While our study focused on analysing the spatiotemporal variation of daily ET using Landsat imagery data, we acknowledge the importance of comparing and contrasting our results with previous studies and literature. Unfortunately, due to the specific scope and focus of our research, direct comparisons with previous studies were limited. However, our findings align with established principles of ET dynamics, emphasizing the influence of climatic factors, land use, and land cover on ET patterns. Future research efforts could further explore the nuances of ET variation and validate our findings against existing literature to enhance the robustness of our conclusions.

The application of the Modified Priestley-Taylor (MPT) model, while a powerful tool for estimating evapotranspiration (ET) and understanding hydrological processes, is subject to various sources of error and uncertainty. These sources can arise from both model assumptions and input data, necessitating careful consideration and validation to ensure the reliability and accuracy of the results. One significant source of uncertainty in the MPT model stems from its reliance on meteorological data, including air temperature. Variations in the quality and spatial resolution of meteorological data can introduce errors into the model output, particularly in regions with heterogeneous climatic conditions or limited ground-based monitoring stations. To mitigate this uncertainty, efforts are made to obtain high-quality meteorological data from reliable sources, such as reanalysis datasets. The authors had access to observed datasets from ground stations, but they aimed to develop a method capable of predicting ET values without relying on any ground observation data. Hence, reanalysis data were used in this study.

Another potential source of error lies in the parameterization of the MPT model itself, including the estimation of model coefficients and calibration constants. These parameters are often derived from empirical relationships or field measurements, introducing inherent uncertainties into the model simulations. To address this challenge, only those constants and calibration processes were involved and used in our study which are previously used by several researchers (References of each were given in material and methods section of the manuscript). Furthermore, efforts are made to calibrate the model using observed ET data or independent validation datasets of lysimeter, thereby improving the accuracy of the simulated ET values.

Various researchers were involved in the use of Landsat 8 and 9 datasets and its relationship with ET in MPT study^47,78–83^. Kerr et al.^84^ and Lo et al.^85^ conducted ET estimations solely using NDVI data, whereas Srivastava et al.^86^ established a correlation between integrated NDVI and plant transpiration. Danodia et al^79^ tested S-SEBI model for estimating crop evapotranspiration (ET_c_) using Landsat-8 data, proved to be effective in estimating and monitoring ET_c_ or consumptive water use over a large area in North India. The results (r = 0.85, RMSE = 0.026, NSME = 0.602 and d = 0.86) aligned with current study. potential evapotranspiration (ET_p_) and single crop coefficient (K_c_) can be accurately calculated using Landsat 8 and Sentinel-2 data through the energy balance equation^78^. The same relationship was also concluded by Khaldi et al.^47^, Paul et al.^80^, Aryalekshmi et al.^83^, Guerschman et al.^87^. Guerschman et al.^87^ calibrated CMRSET model to estimate daily ET_a_ observed at the flux towers with a relative RMSE/R^2^ ranging between 0.15/0.96 (with Sentinel-2) to 0.26/0.93 (VIIRS), furthermore, our research findings closely correspond with one another. The current study has examined the association between ET and NDVI, confirming a significant positive correlation. This finding supports our assertion that higher ET values correlate with lower LST values, and vice versa. Kumar et al.^88^ evaluated the applicability of the simplified surface energy balance index (S-SEBI) method for estimating spatially distributed daily evapotranspiration (ET) using crop coefficient-based coupled Hargreaves–Samani for paddy and potato crop. Our finding shows as promising resulting with higher R-squared and lower RMSE value.

The validation of the Modified Priestley-Taylor (MPT) model for other crops and land surfaces by utilizing readily available EEFlux products. These products provided valuable satellite-based ET estimates that served as independent datasets for validating the performance of the MPT method across diverse land covers and crop types beyond those covered by ground-based measurements^89–92^. The results obtained from comparing the MPT model outputs with EEFlux-derived ET estimates were incorporated into the manuscript, providing additional insights into the model's performance across various agricultural and land use settings. This comprehensive validation approach, which combines ground-based measurements with satellite-derived data, strengthens the reliability and applicability of the MPT model for estimating evapotranspiration in different environmental contexts.

The precise estimation of evapotranspiration (ET) using the modified Priestley-Taylor (MPT) model holds significant implications for agricultural water management strategies. By accurately quantifying ET, farmers and water resource managers can make more informed decisions regarding irrigation scheduling, optimizing water use efficiency, and mitigating water stress in agricultural fields. The MPT model's ability to capture the nuances of ET dynamics, as demonstrated in our study, provides a valuable tool for assessing crop water requirements and guiding irrigation practices. Moreover, by understanding spatiotemporal variations in ET, stakeholders can develop targeted interventions to address water scarcity challenges and enhance agricultural productivity sustainably. Overall, our findings contribute to advancing precision agriculture techniques and supporting evidence-based water management policies, ultimately fostering more resilient and sustainable agricultural systems.

## Conclusion

This research offers valuable insights into the intricate dynamics of daily evapotranspiration (ET) and energy fluxes within the agricultural domain of Udham Singh Nagar district, Uttarakhand, India. The study provides land cover and land classification map of the study area during the rabi season of 2022–23, which gives insights of the prevailing topographical, environmental, and agricultural conditions during the study period. The study also provides the spatiotemporal variation of NDVI and ET values over the Udham Singh Nagar district, which can be utilized for water budgeting, water productivity and irrigation scheduling of the different crops. The finding shows December, characterized as one of the coldest months, experiences limited thermal conditions that suppress the ET process. Subsequently, as temperatures gradually ascend, the ET process commences a gradual intensification, towards the month of April. Therefore, Udham Singh Nagar shows highest ET during April month due to optimal hydrothermal condition.

Through rigorous validation against lysimetric data, particularly focusing on chickpea cultivation, the Modified Priestley-Taylor (MPT) model demonstrates commendable concordance, underscored by an impressive R^2^ value of 0.71. Moreover, the model’s robust performance is evident across various metrics, including the Nash–Sutcliffe Model Efficiency (NSME) and the Agreement Index (d), affirming its accuracy in capturing ET dynamics. The temporal evolution of daily ET values, validated with a robust R^2^ value of 0.84, elucidates a compelling cadence intricately influenced by climatic factors. Notably, the detailed analysis of ET variations reveals a progressive trend mirroring the shifting climatic dynamics across different seasons. This understanding of spatiotemporal variations in energy fluxes and daily ET rates enhances our comprehension of agroclimatic dynamics, offering invaluable insights for informed water resource management strategies in Udham Singh Nagar and analogous agricultural landscapes. In conclusion, this study not only validates the efficacy of the MPT model in ET estimation but also contributes substantively to the broader understanding of agricultural hydrology and climatic influences.

## Limitations

However, it is essential to acknowledge existing limitations and outline future prospects, particularly regarding the generalizability of the model to other regions and different crops. In regions with similar environmental conditions and land use patterns to the study area, the MPT model may be directly transferable with minimal adjustments. However, when extending the model's application to regions with distinct agro-climatic contexts, recalibration and recalculation of model coefficients may be necessary to account for differences in meteorological parameters, soil properties, and crop characteristics. For instance, while the MPT model relies on temperature data as a key input parameter to estimate ET, its applicability to hilly regions is limited due to the significant influence of elevation on temperature variations. In hilly terrains, elevation gradients can induce substantial changes in temperature, resulting in spatially heterogeneous thermal conditions across the landscape. Consequently, the MPT model's performance may be compromised in such areas, as it may not adequately capture the nuanced temperature dynamics associated with elevation changes. In summary, while the MPT model exhibits utility in estimating ET in flat or gently sloping agricultural landscapes with homogeneous surface conditions, its applicability to hilly regions necessitates careful consideration of topographic influences and potential recalibration efforts. Recognizing the inherent limitations of the model in mountainous terrains underscores the importance of tailored approaches and alternative modeling frameworks for accurate ET estimation in such environments.

The validation and performance assessment of the MPT model were conducted using lysimetric data specific to chickpea crop. It doesn’t mean that the MPT model is unable to estimate the ET values of other crops. It can estimate values of different crops pertaining in the study area such as wheat, sugarcane etc. However, to check the reliability of the model over different crop types with varying physiological characteristics and water use patterns demands further investigation. Another limitation of this method is that unavailability of finer resolution data such as daily or weekly. Finer temporal resolutions, such as daily or weekly assessments, may provide deeper insights into short-term variability and crop water requirements.

## Future prospects

Future research endeavours could focus on conducting transferability studies to assess the model's performance across diverse agro-climatic regions and cropping systems. Comparative analyses in contrasting environments would elucidate the model's robustness and identify potential limitations. Additionally, altering the model parameters to different crop species through crop-specific calibration could enhance its accuracy and applicability across a broader spectrum of agricultural landscapes. Apart from these, leveraging advanced remote sensing techniques and high-resolution datasets can further refine the spatial and temporal representation of ET dynamics, facilitating broader-scale applications and monitoring.

## Data Availability

The datasets generated during analysed of the current study are not publicly available due to institution policy but are available from the corresponding author (Dinesh Kumar Vishwakarma) on reasonable request.
